# Co-management strategies for acute myeloid leukemia patients in the community setting

**DOI:** 10.3389/fonc.2022.1060912

**Published:** 2022-12-12

**Authors:** Christopher Benton, Michael R. Grunwald, Hana Safah, Margaret Kasner

**Affiliations:** ^1^ Rocky Mountain Cancer Centers, US Oncology Network, Denver, CO, United States; ^2^ Department of Hematologic Oncology and Blood Disorders, Levine Cancer Institute, Atrium Health, Charlotte, NC, United States; ^3^ Tulane Cancer Center, Tulane University School of Medicine, New Orleans, LA, United States; ^4^ Department of Medical Oncology, Sidney Kimmel Cancer Center, Thomas Jefferson University, Philadelphia, PA, United States

**Keywords:** acute myeloid leukemia, community, diagnosis, management, treatment, hematopoietic cell transplantation

## Abstract

The treatment landscape for acute myeloid leukemia (AML) has changed substantially in recent years. The introduction of newer therapies, including oral agents, less myelosuppressive agents, and parenteral regimens suitable for outpatient administration, has made it feasible for select patients to receive therapy in the outpatient setting and in community practices. Thorough patient evaluation (including molecular testing), planned supportive care (eg, transfusion support, antimicrobial prophylaxis), and vigilant patient monitoring (for tumor lysis syndrome and adverse events) by a multidisciplinary team are required for successful management of patients both in the community and at specialized leukemia centers. Some patients are unable or unwilling to travel to larger academic centers for treatment, and treatment of AML in the community setting may have potential advantages compared to less conveniently located academic/leukemia centers. This includes reduction of financial hardship for patients and their families and often better opportunities for family/caregiver support. Additionally, partnership between community practices and academic/leukemia centers is often crucial to optimizing AML management for many patients, as collaboration may facilitate access to additional expertise and trials, multidisciplinary teams for supportive care, easier transition to hematopoietic cell transplantation, and access to sophisticated molecular testing. In this review, we discuss AML treatment and management in the community setting, available therapies, and circumstances in which a referral to and co-management with an academic/leukemia center is more strongly recommended.

## Introduction

Acute myeloid leukemia (AML) is an aggressive hematologic malignancy primarily affecting older individuals (median age at diagnosis is 68 years), many of whom have significant comorbidities. Five-year AML survival decreases sharply with increasing age, from 59% for ages <50 years to 8% for ages ≥65 years (1). Although intensive induction chemotherapy followed by hematopoietic cell transplantation (HCT) remains a standard of care for most medically fit patients treated with curative intent, the management of AML has been enriched in recent years by the approval of several therapies indicated for specific, but often overlapping, subpopulations, and the expansion of allogeneic HCT to more patients. Multidisciplinary teams, consisting of advanced practice providers, nurses, pathologists, pharmacists, navigators, and social workers, are thus important for the successful management of patients with AML. Newer therapies have also made it feasible for select patients to receive therapy in the outpatient setting and in community practices, and treatment of AML in the community setting may have potential advantages that include reduction of financial hardship for patients and their families and often better opportunities for family/caregiver support. Herein, we discuss the diagnosis and dynamic management of patients with AML, with emphasis on issues relevant to community practices.

## Diagnosis of AML

The inclusion of fluorescence *in situ* hybridization, karyotyping/cytogenetic, and mutation testing in diagnostic evaluations is important to determine the AML subtype and identify actionable mutations, which can guide treatment decisions. The European LeukemiaNet recommends a diagnostic workup including screening for mutations that define disease and risk categories or are therapeutic targets (2). Mutational testing is recommended at diagnosis and again at relapse (3, 4). Recent studies suggest waiting for key cytogenetic and molecular test results before initiating treatment is generally preferred over early treatment initiation without this information, as the test results may guide treatment decisions (5, 6).

Initial diagnosis commonly occurs in the emergency room or inpatient service in community hospitals. If a challenging diagnosis is encountered or a substantial delay (≥1 week) in key test results is expected, referral from a community practice to an academic or specialized leukemia program should be considered (7). Examples of challenging diagnoses may include biphenotypic acute leukemia, acute leukemia of ambiguous lineage, pure erythroid leukemia, Philadelphia-positive AML, or AML with extramedullary disease only. Regular collaboration with an academic/leukemia center can facilitate faster and more thorough diagnostic workups and provide expertise from experienced hematopathologists. Referral can also help with early evaluation of patients for their potential to participate in a clinical trial and/or undergo allogeneic HCT. In the event referral to an academic/leukemia center is not possible, standard procedures should be in place to ensure proper evaluation and testing of all patients and avoid delays in treatment.

## Treatment selection for AML

Several factors influence treatment decisions for AML, including age, presence of comorbidities, performance status, AML subtype, cytogenetic risk category, mutation profile, patient preferences, and treatment goals (eg, curative intent) (8). While the American Society of Hematology (ASH) recommends medically fit patients receive intensive induction therapy as the optimal path to long-term remission (9), some eligible patients may be better suited to less-intensive regimens to achieve their goals. Recent research also suggests patients’ socioeconomic circumstances may affect treatment decisions and outcomes (10). In the last several years, new therapies representing a spectrum of intensities have been approved for the treatment of various AML subpopulations. Community oncology practices may have limited experience with certain newer therapies due to the low frequency of candidate patients. Ongoing communication with an academic/leukemia center provides an avenue for the discussion of treatment options with other providers, review of medications and provision of recommendations from specialty pharmacists, and provision of educational materials to patients. Participation in a clinical trial should also be considered for all patients.

Conventional frontline induction therapy for AML is typically administered in the inpatient setting because of the need for long and/or multiple infusions and close monitoring for myelosuppression and infection. This is currently the standard-of-care for younger, fit patients in whom HCT is a consideration. However, the approval of new therapies and improvements in supportive care have permitted the administration of less-intensive therapies, as well as some intensive regimens, in the outpatient setting. Preferred outpatient regimens are administered orally or as a short infusion, less myelosuppressive, and tolerable with outpatient management of toxicities. Each patient should be carefully evaluated for their appropriateness for outpatient treatment (11, 12). Considerations include a patient’s age, overall health, distance from the hospital, agreement to alert the center if they experience adverse events (eg, fever), preference for outpatient therapy, and availability of a caregiver to monitor the patient and assist them with transportation to frequent follow-up visits. Outpatient treatment is generally not recommended for patients with high disease burden, high risk for tumor lysis syndrome, and/or poor renal function (13).

Of note, patients with acute promyelocytic leukemia require careful monitoring; however, management of these patients is outside the scope of this article.

## Management of AML

### Management/co-management in the community setting and referral

Treatment and management of AML in the community setting is appropriate for many patients and provides advantages compared with academic/leukemia centers, such as prevention of travel hardship for patients and their families and often better family/caregiver support (7, 14). A quality-of-life questionnaire administered to patients undergoing treatment for AML revealed that family, friends, and community were their most important sources of support (15), emphasizing the role of community management/co-management. However, there are circumstances in which a patient’s referral from a community practice to an academic/leukemia center should be considered (7, 14), and these are outlined in **Table 1**. One recent retrospective database study found that patients treated in hospitals with a high volume of intensive chemotherapy use for AML were less likely to die or be discharged to hospice and were more likely to undergo a bone marrow assessment and to receive anti-infectives than those treated in low-volume centers (16). A separate retrospective database study found lower 1-month mortality and greater 5-year survival for patients managed in academic versus nonacademic centers; patients managed in academic centers were also more likely to receive intensive therapy and proceed to HCT (17). However, it should be noted that community practices vary greatly in their experience, staff size, and facilities/resources, which can impact their ability to provide the support needed to successfully treat and manage/co-manage patients with AML. Clinical trial participation is also recommended, and some trials may allow patients to be monitored in a community practice while periodically visiting an academic/leukemia center.

**Table 1:** Recommendations for considering referral from a community practice to a larger academic/leukemia center.

Therapy that requires supportive care that is not available at the facility (including availability of infusion centers with extended daily and weekend/holiday hours for transfusion support)
Limited experience with a specific therapy
Therapy-related AML or AML-MRC (including secondary AML with an antecedent hematologic disorder)^a^
Relapsed/refractory AML
Consideration for clinical trial participation (especially in patients who can not receive standard chemotherapy regimens)
Younger patients and/or those treated with curative intent who are likely to receive intensive therapy
Patient preference
Possible future candidate for allogeneic HCT; considerations for referral/co-management with transplant centers: Determination of a patient’s eligibility for HCT Facilitation of pre-HCT workup Access to a broader range of protocols Reduced time to HCT

AML, acute myeloid leukemia; AML-MRC, acute myeloid leukemia with myelodysplasia-related changes; HCT, hematopoietic cell transplantation.

a These patients can be co-managed between a community practice and a larger academic/leukemia center. Primary reason for referral is the need for transplant.

Patient management does not need to be administered exclusively at a community practice versus an academic/leukemia center. In some cases, a community oncologist may help diagnose a patient and either initiate treatment under his/her care or transfer the patient to an academic/leukemia center to initiate treatment. In other cases, a patient may be diagnosed and initiate treatment at an academic/leukemia center with a specialized leukemia physician and then establish continued care with a community oncologist. Close collaboration between practices is important for successful management of patients, as it facilitates the availability of additional expertise, multidisciplinary teams, and easier transition to HCT (7). Collaboration between practices can be facilitated in several different ways—*via* phone conversations, secure messaging, in-person consultation between a provider and patient, e-consultation (remote chart review and opinion), and/or virtual consultation. A variety of multidisciplinary team members contribute to the medical management of patients, including physicians, nurses, pharmacists, nutritionists, infectious disease specialists, physical/occupational therapists, and other advanced care practitioners. In addition to the multidisciplinary team’s medical management of patients, nurses, pharmacists, transplant coordinators, and advanced care practitioners can provide patient education and help coordinate logistics, while nurse navigators and social workers provide additional patient and caregiver support. Multidisciplinary team meetings, such as tumor boards, can optimize patient care coordination and decision making for complex cases. Co-management between practices should consider the experience and capabilities of each practice, as well as regional variations in practices. For example, in some instances, referred patients may be required to travel long distances to academic centers for AML treatment. Given this inconvenience, post-consolidation supportive care can be administered in the community setting for many patients, with co-management between practices to give patients access to specialized care. In a retrospective analysis, post-consolidation supportive care at local treatment centers versus quaternary centers reduced the burden of patient travel without compromising outcome (18). The rapid increase in access to telehealth in recent years has further facilitated co-management between practices. With this technology, some aspects of patient care can be overseen by academic/leukemia centers without the need for travel; however, its use is often restricted across state lines.

### Management/co-management in the outpatient setting

Considerations for the co-management of patients in the outpatient setting are provided in **Table 2**. A community practice’s ability to administer therapy and manage patients in the outpatient setting depends on their experience with the agent/regimen, their logistical capabilities to provide adequate supportive care, and the commitment of academic/leukemia centers’ multidisciplinary teams to providing regular co-management support. New technologies to aid in patient care in the outpatient setting may include secure messaging, wearables/home vital sign monitoring, and patient reporting apps for symptoms. However, it should be noted that outpatient management of patients with AML may not be appropriate at some smaller community practices with limited experience, even with co-management support. Open communication *via* phone or email between practices is necessary. The availability of a tumor board that meets regularly to discuss difficult cases and therapy updates is also important.

**Table 2:** Considerations for co-managing patients in the outpatient setting.

Preparation	• Multidisciplinary team, including physicians, nurses, pharmacists, and advanced care practitioners, with an expertise in AML management
Patient selection	Evaluate patient’s age Evaluate patient’s overall health Consider patient’s preferences Evaluate patient’s distance from the treatment center Ensure availability of a caregiver to monitor the patient and transport him/her to scheduled visits
Education	Education of the oncology team on preparing patients and caregivers, patient monitoring, arranging supportive care, and transition to HCT Education of patients and caregivers by a team of physicians, nurses, pharmacists, advanced care practitioners, nurse navigators, and social workers Education of patients and caregivers on concerning signs and symptoms to aid in the quick identification and management of adverse events
Monitoring	Coordination of supportive care to be administered following treatment – 7 + 3–based regimens: commitment to 7 days of continuous chemotherapy – CPX-351: commitment to 5 consecutive days of therapy/management (treatment and supportive care on Days 1, 3, and 5, and supportive care on Days 2 and 4) – Venetoclax plus HMAs or LDAC: commitment to continuous outpatient therapy Careful monitoring of patients for adverse events Ability to rapidly identify and initiate treatment for neutropenic fever Availability of an infusion center with extended daily and weekend/holiday hours for transfusion support Appropriate cardiac workup Central IV access placement Survivorship and palliative care services

AML, acute myeloid leukemia; HCT, hematopoietic cell transplantation; HMA, hypomethylating agent; LDAC, low-dose cytarabine; IV, intravenous.

Successful implementation of outpatient treatment requires quick and easy patient access to providers in case complications arise (19). Planned supportive care and careful monitoring of patients for adverse events is crucial for success in outpatient management, in both community and academic/leukemia center settings. Patients and caregivers should receive education on concerning signs and symptoms to aid in the quick identification and management of adverse events. Studies suggest outpatient management after intensive induction chemotherapy is often feasible and safe (20). Later lines of therapy in relapsed/refractory AML are also commonly administered in the outpatient setting. However, many patients may still be hospitalized during or after induction therapy for closer management of cytopenias and other complications (21).

Regimens such as venetoclax (a once-daily oral agent) plus either a hypomethylating agent (HMA) or low-dose cytarabine (LDAC) can be administered on an outpatient basis at community practices with guidance/consultation in conjunction with academic/leukemia centers. A bone marrow biopsy should be obtained early (after the first cycle, when blasts are cleared), and patients must be closely monitored for myelosuppression throughout treatment.

Although conventional intensive chemotherapy is often administered in the inpatient setting, CPX-351, a dual-drug liposomal encapsulation of daunorubicin and cytarabine at a synergistic 1:5 molar ratio, can be administered as induction or consolidation in the outpatient setting (13, 19, 22–24). This is possible due to sequestering of the active drugs within the liposome, thereby limiting immediate toxicity, and its dosing schedule (discrete 90-minute infusions on Days 1, 3, and 5 for the first induction and Days 1 and 3 for the second induction and consolidation) (25). Due to the potential for CPX-351 to cause prolonged myelosuppression, outpatient administration is only recommended for community practices that have experience with this therapy and/or frequent communication with an academic/leukemia center and planned transfusion support (ideally on the same day as chemotherapy, if necessary). Notably, patients who are referred for transplant and co-managed between an academic/leukemia center and an experienced community practice may be the best candidates for such outpatient approaches.

### Management/co-management in patients who are candidates for transplant

Intensive therapy followed by allogeneic HCT is currently the most well-established treatment pathway with curative intent for AML. With HLA-matched related or unrelated donor transplantation, as well as haploidentical donor transplantation using the posttransplant cyclophosphamide approach (26), most patients in need of HCT now have a donor. Alternative donor sources, such as unrelated umbilical cord blood and mismatched unrelated donors, can also be considered (26). Age itself is no longer a barrier to HCT and does not impact non-relapse mortality or the frequency of relapse or graft-versus-host disease (27).

Patients should be referred early to a transplant center (which can be part of the same academic/leukemia center providing general leukemia guidance) for consultation if there is a reasonable chance they may become an HCT candidate. Referral to or co-management with transplant centers can help with determination of a patient’s eligibility for HCT, facilitation of pre-HCT workup, access to a broad range of protocols, and reduced time to HCT. Early referral for HLA typing and donor identification is an important consideration for a seamless transition to HCT and can be accomplished by any practice through contacting organizations such as bethematch.org. Additionally, early HLA typing permits a quick transition to HCT, which several studies suggest may be the best approach for primary induction failure or first relapse (28–31).

Transplant centers can perform workups for HCT while patients are still undergoing therapy in community practices and can provide specialized leukemia guidance to community oncologists. As with outpatient therapy, successful co-management of HCT candidates requires commitment of academic/leukemia centers’ multidisciplinary team members to providing regular support, with open communication between practices. Once patients undergo HCT, they typically remain under the care of transplant centers; nevertheless, periodic follow-up from community practice providers is valuable.

## Use of AML therapies in the community setting

The AML treatment landscape has expanded greatly in recent years, with available treatments now representing a range of intensities and indications for various AML subpopulations. In a recent analysis, practice patterns of community healthcare providers differed from those of the experts in AML (32). This may be partly due to community healthcare providers having less experience with newer therapies, in which case, co-management with academic/leukemia centers may provide opportunities for better patient care. **Supplementary Table S1** shows the results of key studies and provides management considerations for agents commonly used for the treatment of AML. Cytogenetic and molecular testing is necessary for establishing patient eligibility for targeted agents and should be performed in a timely manner.

## Case studies

### Patient 1

A 73-year-old female was admitted to a community oncology center and diagnosed with *de novo* AML (white blood cell count: 5.4×10^9^/L with 39% circulating blasts; absolute neutrophil count [ANC]: 0.6×10^9^/L; platelet count: 31×10^9^/L; hemoglobin: 6.9 g/dL). Molecular testing did not identify a targetable mutation, and targeted therapy was thus not considered. The patient had significant comorbidities, including diabetes, systolic heart failure with an ejection fraction of 35%, and stage 3 chronic kidney disease. Intensive chemotherapy was not considered due to the presence of these significant comorbidities; however, she was reasonably high functioning despite her comorbidities and received venetoclax plus azacitidine rather than best supportive care in hopes of achieving remission and prolonging survival. The selection of treatment over best supportive care is consistent with recommendations from ASH for older adults with AML (9).

The patient preferred to be treated locally in the outpatient setting; this was accomplished through close patient monitoring and regular communication between the community practice and a larger leukemia center. The patient was admitted to the hospital due to prolonged myelosuppression. She was monitored carefully with daily laboratory testing for 14 days, until her blood counts recovered to a clinically significant threshold (4). Given her low ANC, antimicrobial prophylaxis with CYP3A inhibitors (ciprofloxacin and voriconazole) and acyclovir was initiated. Due to drug-drug interactions with CYP3A inhibitors, the venetoclax dose was reduced; however, dose interruptions were not necessary. Although many centers administer venetoclax plus HMA or LDAC in the outpatient setting, myelosuppression is common and can be severe; blood counts should be carefully monitored throughout treatment, and supportive care should be administered when needed (33). The patient was also monitored carefully for signs of tumor lysis syndrome, although its reported frequency is low in AML (33).

The patient achieved complete remission with measurable residual disease (MRD) negativity after 2 cycles of therapy. Although some patients who receive lower/intermediate-intensity therapy with venetoclax plus azacitidine may be HCT candidates, this patient was not a candidate due to her comorbidities. Maintenance therapy with CC-486 was considered; however, after the earlier hospitalization her blood counts fully recovered and she tolerated venetoclax plus azacitidine without dose interruptions, and so she continued this regimen to prolong remission.

### Patient 2

A 64-year-old male with prior myelodysplastic syndrome was admitted to a community hospital and evaluated by an oncology practice (white blood cell count: 0.7×10^9^/L with 8% circulating blasts; ANC: 0.3×10^9^/L; platelet count: 19×10^9^/L; hemoglobin: 7.1 g/dL). Based on his antecedent myelodysplastic syndrome, the patient was diagnosed with AML with myelodysplasia-related changes; molecular testing identified no targetable mutations, but cytogenetic testing revealed a complex karyotype. The patient had hypertension, but it was well controlled and he was otherwise relatively healthy. He was therefore considered a candidate for intensive chemotherapy in hopes of achieving durable remission and proceeding to HCT and was referred to the transplant center of a larger academic institution for a work-up.

Recommendations from ASH suggest eligible older adults receive intensive therapy with a curative intent (9). After discussion between practices, the patient received CPX-351 induction; this decision was based on evidence that treatment with conventional chemotherapy leads to poorer outcomes versus CPX-351 in patients with unfavorable clinical features, such as AML with an antecedent hematologic disease (25, 34). The decision was also supported by current treatment guidelines from the National Comprehensive Cancer Network and the European Society for Medical Oncology (4, 35). Targeted agents were not considered since he had no targetable mutations.

The patient was concerned about frequent travel, so treatment was administered at his community practice in the inpatient setting, with close communication between practices to help mitigate complications. The patient experienced prolonged myelosuppression requiring transfusion support and developed neutropenic fever. This experience was consistent with the phase 3 study of CPX-351 versus the 7 + 3 chemotherapy regimen, in which CPX-351 prolonged myelosuppression but otherwise had a safety profile generally similar to that of 7 + 3 (25). The patient also developed mild fatigue and mouth sores. All adverse events resolved with conservative management.

The patient achieved complete remission without MRD and subsequently received 1 cycle of CPX-351 consolidation at his community practice in the outpatient setting. The outpatient setting was chosen for consolidation out of convenience and because of his previous experience with CPX-351 induction therapy. The patient was subsequently transitioned to the transplant center and proceeded to allogeneic HCT while still in MRD-negative remission. The initial post-HCT follow-up, which requires careful monitoring for infection or graft-versus-host disease, was carried out at the transplant center, after which long-term follow-up was shared between the community practice and transplant center to reduce travel.

### Patient 3

A 68-year-old male was admitted to the hospital with elevated white blood cell count (32×10^9^/L with 47% circulating blasts) and low hemoglobin (9.2 g/dL) and platelet counts (47×10^9^/L) approximately 1 year after achieving complete remission with 7 + 3 chemotherapy for *de novo* AML. The patient was diagnosed with relapsed AML, and molecular testing upon relapse led to the discovery of a new *IDH2* mutation. Mutational testing should be repeated at each relapse, as it may identify new targetable mutations or appropriate clinical trials (3, 4). Although the patient had previously been treated with intensive 7 + 3 chemotherapy, he was not considered a candidate for intensive salvage therapy due to the development of cardiac complications. Due to the presence of a new *IDH2* mutation, the patient received enasidenib, with treatment administered in the outpatient setting through his community practice.

After a month on enasidenib treatment, the patient reported fever and dyspnea. Differentiation syndrome was suspected, and the patient was hospitalized and given dexamethasone for 3 days. The patient recovered and was able to continue receiving enasidenib in the outpatient setting with careful monitoring. Differentiation syndrome, which was observed in 14% to 25% of patients treated in clinical trials with the IDH inhibitors enasidenib and ivosidenib, can be life-threatening if not treated; patients receiving these agents should be educated on common symptoms of differentiation syndrome so they can promptly alert their oncology team and receive treatment (36, 37).

The patient quickly achieved transfusion independence, which converted to complete remission with incomplete hematologic recovery after 2 additional cycles. The case was discussed with a transplant center, but the patient was not considered a candidate for allogeneic HCT at that time due to psychosocial reasons; instead, he was continued on enasidenib.

## Discussion

Patients with AML may be treated in the community setting by hematologists/oncologists with varying degrees of comfort regarding the changing landscape of AML therapy. Some cases may require referral to academic/leukemia centers, while other cases may be treated locally. Additionally, survivorship and palliative care for patients in the community setting allows for better opportunities for emotional and financial support and better family/caregiver support, which can be crucial for the patient’s quality of life. In the community setting, patients should be managed in a detail-oriented, multi-disciplinary fashion. Where possible, patients should be considered for clinical trials. AML management in the community can often be optimized by close collaboration between community practices and academic/leukemia and transplant centers, thereby facilitating optimal patient care for a challenging-to-treat diagnosis.

## Data availability statement

The original contributions presented in the study are included in the article/**Supplementary Material**. Further inquiries can be directed to the corresponding author.

## Author contributions

All authors contributed to the identification of topics for inclusion, evaluation of literature, and writing and/or critical review of this manuscript, and all authors approved the final version of this manuscript for publication.
