# Supplementary material for: Co-management strategies for acute myeloid leukemia patients in the community setting
Source: Front Oncol. 2022 Dec 12;12:1060912. doi: 10.3389/fonc.2022.1060912 (PMC9791081; doi:10.3389/fonc.2022.1060912)
Supplement: Supplementary file 1 [file DataSheet_1.pdf]

Supplementary Appendix

Co-management Strategies for Acute Myeloid Leukemia Patients in the Community Setting

Christopher Benton, Michael R. Grunwald, Hana Safah, Margaret Kasner

Supplementary Table 1. Commonly Used Treatment Options for Patients With AML

Agent/ regimen	AML population(s)	Administration schedule	Key efficacy outcomes	Key safety outcomes/concerns	Ability for outpatient administration	Key management considerations
**Intensive chemotherapy**						
7+3[a]	Newly diagnosed and R/R AML eligible for intensive therapy	• Cytarabine 100-200 mg/m^2/day for 7 days plus daunorubicin 60-90 mg/m^2 or idarubicin 12 m/m^2 on Days 3 to 5	Adults with newly diagnosed AML:<br>• mOS: 9.1-24 mo (1-3)<br>• CR+CRi: 50%-82% (1-3)	• May induce significant myelosuppression; neutropenic fever is common during induction cycle (4)<br>• Potential for mucositis, GI events, and infection	• Due to the multiple-day infusion schedule, 7+3 is typically restricted to the inpatient setting with prolonged hospitalization (5)	• Immediate management of neutropenic fever is critical
HiDAC	• Newly diagnosed and R/R AML eligible for intensive therapy<br>• Post-remission therapy	• Cytarabine 2 g/m^2 every 12 hours for 6 days or 3 g/m^2 every 12 hours for 4 days; may be given with idarubicin or daunorubicin and etoposide<br>• Dose reduction may be necessary for patients >60 years	Adults ≤60 years with newly diagnosed AML:<br>• RFS at 5 years: 49% (6)<br>• CR: 71% (6)	• May induce significant myelosuppression; neutropenic fever is common during induction cycle (4)<br>• Potential for mucositis, GI events, and infection	• Due to the multiple-day infusion schedule, HiDAC is commonly administered in the inpatient setting (5)<br>• However, HiDAC consolidation may be given in an outpatient setting	• Immediate management of neutropenic fever is critical
FLAG-Ida	• Newly diagnosed and R/R AML +/− venetoclax	• Fludarabine 30 mg/m^2 + cytarabine 2 g/m^2 for 5 days plus idarubicin 10 mg/m^2 on Days 1 to 3 plus granulocyte colony-stimulating factor 300 µg/m^2 on Days −1 to 5	Newly diagnosed or R/R AML or MDS:<br>• CR+CRi: 95% (7)<br>• Patients proceeding to HCT: 16% (7)<br><br>Newly diagnosed or R/R AML in combination with venetoclax:<br>• CR+CRi in newly diagnosed AML: 90% (8)<br>• CR+CRi in R/R AML: 61% (8)	• Most common AEs include myelosuppression, nausea, vomiting, diarrhea, and mucositis (7)	• Due to the multiple-day infusion schedule, FLAG-Ida is commonly administered in the inpatient setting	• Generally tolerable, with a risk of myelosuppression

			• Patients proceeding to HCT: 56% (8)<br>• 1-year OS in patients proceeding to HCT: 87% (8)			
CPX-351 (dual-drug liposomal encapsulation of daunorubicin and cytarabine at a synergistic 1:5 molar drug ratio)	Newly diagnosed therapy-related AML or AML-MRC in adults and pediatric patients aged ≥1 year who are eligible for intensive therapy	• CPX-351: 100 units/m^2 (daunorubicin 44 mg/m^2 and cytarabine 100 mg/m^2) on Days 1, 3, and 5 (first induction) or Days 1 and 3 (second induction)	Adults 60-75 years:<br>• mOS vs 7+3: 9.33 vs 5.95 mo (HR = 0.70) (9)<br>• CR+CRi vs 7+3: 48% vs 33% (9)<br>• Patients proceeding to HCT vs 7+3: 35% vs 25% (9)<br>• 3-year OS landmarked from the date of HCT vs 7+3: 56% vs 23% (9)	• Most common grade 3-4 AEs include myelosuppression, febrile neutropenia, pneumonia, and hypoxia (10)	• Administered as a 90-minute infusion, enabling administration in an outpatient setting (11-15)	• Administered in inpatient or outpatient setting, depending on center experience and logistics<br>• Patients receiving outpatient treatment may need to be hospitalized for recovery of complications (unplanned or planned)<br>• Frequent monitoring and supportive care are required for risk of prolonged myelosuppression, infection, or other complications
**Low-intensity therapy**						
Azacitidine (HMA)	Newly diagnosed and R/R AML in older adults and those who have comorbidities that preclude the use of intensive induction chemotherapy	• Azacitidine 75 mg/m^2/day for 7 consecutive days per 28-day treatment cycle	• mOS vs conventional care: 10.4 vs 6.5 mo (HR = 0.85; *P* = 0.1009) (16)<br>• CR+CRi vs conventional care: 28% vs 25% (16)	• Most common AEs include nausea and myelosuppression (16)	• Patients can often remain outpatients throughout their HMA course (17,18)	• Generally tolerable
Decitabine (HMA)	Newly diagnosed and R/R AML in older adults and those who have comorbidities that preclude the use of intensive induction chemotherapy	• Decitabine 20 mg/m^2/day for 5 consecutive days every 4 weeks	• mOS vs treatment of choice: 7.7 vs 5.0 mo (HR = 0.82; *P* = 0.037) (19)<br>• CR+CRp vs treatment of choice: 18% vs 8% (19)	• Most common AEs include myelosuppression and febrile neutropenia (19)	• Patients can often remain outpatients throughout their HMA course (17,18)	• Generally tolerable

**Lower/intermediate-intensity therapy**						
Venetoclax (small-molecule inhibitor of anti-apoptotic Bcl-2)[b] combinations	In combination with HMAs or LDAC for newly diagnosed AML in adults aged ≥75 years or who have comorbidities that preclude the use of intensive induction chemotherapy (20,21)	Venetoclax plus azacitidine:<br>• Venetoclax 400 mg plus azacitidine 75 mg/m^2 after initial venetoclax dose ramp-up<br><br>Venetoclax plus LDAC:<br>• Venetoclax 600 mg/day plus LDAC 20 mg/m^2/day on Days 1 to 10; 28-day cycles	Venetoclax plus azacitidine:<br>• mOS vs azacitidine: 14.7 vs 9.6 mo (HR = 0.66; *P* <0.001) (22)<br>• CR+CRi vs azacitidine: 66% vs 28% (22)<br>• Patients proceeding to HCT vs azacitidine: <1% vs <1% (22)<br><br>Venetoclax plus LDAC:<br>• mOS vs LDAC: 7.2 vs 4.1 mo (HR = 0.75; *P* = 0.11) (23)<br>• CR+CRi vs LDAC: 48% vs 13% (23)<br>• Patients proceeding to HCT vs LDAC: 0% vs 0% (23)	Venetoclax plus azacitidine:<br>• Most common AEs include myelosuppression, febrile neutropenia, and GI events (22)<br><br>Venetoclax plus LDAC:<br>• Most common AEs include myelosuppression febrile neutropenia, GI events, and hypokalemia (23)	• Can be administered in the outpatient setting with close monitoring for myelosuppression<br>• In a retrospective analysis in patients with AML who received venetoclax plus HMAs either as frontline or R/R therapy, outpatient ramp up of venetoclax was safe with no evidence of clinical TLS (24)	• Prophylactic measures for TLS prevention are necessary<br>• Close communication with an academic/ leukemia center may be valuable for patients who are being treated in the community setting due to risk of severe myelotoxicity (25,26)<br>• To avoid unnecessary dose interruptions or dose reductions, close monitoring and supportive care is recommended (13)
**Moderate-intensity therapy**						
Cladribine + LDAC +/- venetoclax	Newly diagnosed AML in older adults	• Cladribine 5 mg/m^2 on Days 1 to 5 followed by LDAC 20 mg twice daily on Days 1 to 10<br>• Venetoclax 400 mg on Days 1 to 21	Cladribine + LDAC + venetoclax:<br>• mOS: not reached (27)<br>• mEFS: not reached<br>• CR+CRi: 93% (27)<br><br>Cladribine + LDAC:<br>• mOS: 13.8 mo (28)<br>• CR+CRi: 68% (28)<br>• Patients proceeding to HCT: 15% (28)	Cladribine + LDAC + venetoclax:<br>• Most common grade 3-4 AEs include neutropenic fever and pneumonia (27)<br><br>Cladribine + LDAC:<br>• Most common AEs include myelosuppression, infection, elevated total bilirubin, rash, and nausea (28)	• Can be administered in the outpatient setting with close monitoring	• Generally tolerable

**Targeted therapy**						
Midostaurin (FLT3 inhibitor)	In combination with 7+3 chemotherapy for the treatment of adults with newly diagnosed, *FLT3*-mutated AML	• Midostaurin 50 mg twice daily on Days 8 to 21 plus 7+3	• mOS vs 7+3: 74.7 vs 25.6 mo (HR = 0.78; *P* = 0.009) (29)<br>• CR vs 7+3: 59% vs 54% (29)<br>• Patients proceeding to HCT vs 7+3: 59% vs 55% (29)	• Most common grade 3-5 AEs include myelosuppression, febrile neutropenia, and infection (29)	• Typically administered in the inpatient setting due to combination with 7+3	• Generally tolerable, with a risk of gastrointestinal distress
Gilteritinib[c] (FLT3 inhibitor)	Adults with R/R *FLT3*-mutated AML	• Gilteritinib 120 mg once daily; 28-day cycles	• mOS vs salvage chemotherapy: 9.3 vs 5.6 mo (HR = 0.64; *P* <0.001) (30)<br>• CR+CRi vs salvage chemotherapy: 34% vs 15% (30)<br>• Patients proceeding to HCT vs salvage chemotherapy: 26% vs 15% (30)	• Most common grade 3-4 AEs include febrile neutropenia and myelosuppression (30)	• Generally administered in the outpatient setting	• Generally tolerable
Ivosidenib[d] (IDH1 inhibitor)	Adults with R/R *IDH1*-mutated AML and those with newly diagnosed *IDH1*-mutated AML who are aged ≥75 years or ineligible for intensive chemotherapy	• Ivosidenib 500 mg once daily; 28-day cycles	<u>R/R AML:</u><br>• mOS: 8.8 mo (31)<br>• CR+CRi: 30% (31)<br><br><u>Newly diagnosed AML:</u><br>• mOS: 12.6 mo (32)<br>• CR+CRi: 42% (32)	• Most common grade 3-4 AEs include prolongation of QT interval, differentiation syndrome, and leukocytosis	• Generally administered in the outpatient setting	• Generally tolerable, with a low risk of myelosuppression
Ivosidenib (IDH1 inhibitor) + azacitidine	Adults with previously untreated *IDH1*-mutated AML ineligible for intensive chemotherapy	• Ivosidenib 500 mg + azacitidine 75 mg/m^2 body surface area once daily for 7 days; 28-day cycles	• mEFS vs azacitidine: 22.9 vs 4.1 mo (HR = 0.33) (33)<br>• mOS: 24.0 vs 7.9 mo (33)<br>• CR+CRp: 53% vs 18% (33)<br>• ORR: 62% vs 19% (33)	• Most common grade 3-4 AEs included febrile neutropenia, anemia, neutropenia, thrombocytopenia, and pneumonia (33)	• Generally administered in the outpatient setting	• Generally tolerable, with a low risk of myelosuppression
Enasidenib[e] (IDH2 inhibitor)	Adults with R/R *IDH2*-mutated AML	• Enasidenib 100 mg once daily; 28-day cycles	• mOS: 9.3 mo (34)<br>• ORR: 40% (34)	• Most common grade 3-4 AEs include hyperbilirubinemia,	• Generally administered in the outpatient setting	• Generally tolerable, with a low risk of myelosuppression

			• Patients proceeding to HCT: 10% (34)	differentiation syndrome, and myelosuppression (34)		
Gemtuzumab ozogamicin (anti-CD33 antibody conjugate)	• Monotherapy or in combination with 7+3 for adults with newly diagnosed, CD33-positive AML<br>• Monotherapy for R/R AML<br>• Monotherapy for older adults with newly diagnosed AML	• Gemtuzumab ozogamicin 3 mg/m^2 on Days 1, 4, 7 plus 7+3	• EFS at 2 years vs 7+3: 41% vs 17% (35)<br>• CR+CRp vs 7+3: 81% vs 75% (35)<br>• Patients proceeding to HCT vs 7+3: 4% vs 4% (35)	• Most common grade 3-4 AEs include thrombocytopenia and hemorrhage (35)	• Monotherapy: generally administered in the outpatient setting with close monitoring for blood counts and liver function<br>• In combination with 7+3: generally administered in the inpatient setting	• Generally tolerable, with a risk of thrombocytopenia or veno-occlusive disease
Glasdegib (Hedgehog signaling pathway inhibitor)	In combination with LDAC for adults with newly diagnosed AML aged ≥75 years or who have comorbidities precluding the use of intensive chemotherapy	• Glasdegib 100 mg once daily; 28-day cycles	• mOS vs LDAC: 8.8 vs 4.9 mo (36)<br>• CR vs LDAC: 17% vs 2% (36)	• Most common grade 3-4 AEs include anemia and febrile neutropenia (36)	• Generally administered in the outpatient setting	• Generally tolerable
CC-486 (oral azacitidine)	Adults with AML who achieved first CR or CRi following intensive chemotherapy and are unable to complete intensive curative therapy	• CC-486 300 mg once daily on Days 1 to 14 of a 28-day cycle	• mOS vs placebo: 24.7 vs 14.8 mo (37)	• Most common grade 3-4 AEs include neutropenia and thrombocytopenia (37)	• Generally administered in the outpatient setting	• Generally tolerable, with a risk of gastrointestinal toxicity and myelosuppression

Abbreviations: AE, adverse event; AML, acute myeloid leukemia; AML-MRC, acute myeloid leukemia with myelodysplasia-related changes; Bcl-2, B-cell lymphoma 2; CR, complete remission; CRi, complete remission with incomplete neutrophil or platelet recovery; CRp, complete remission with incomplete platelet recovery; DFS, disease-free survival; EFS, event-free survival; FLAG-Ida, fludarabine, HiDAC, and granulocyte colony-stimulating factor with idarubicin; HCT, hematopoietic cell transplantation; HMA, hypomethylating agent; HR, hazard ratio; LDAC, low-dose cytarabine; mOS, median overall survival; MDS, myelodysplastic syndrome; MRD, measurable residual disease; ORR, overall response rate; OS, overall survival; R/R, relapsed/refractory; TLS, tumor lysis syndrome.

[a]High-dose cytarabine (HiDAC)–containing regimens represent an alternative intensive chemotherapy approach. These include the FLAG/FLAG-Ida and CLAG/CLAG-M (cladribine, HiDAC, and granulocyte colony-stimulating factor, with or without mitoxantrone) regimens. Also, as another alternative intensive chemotherapy approach, newer drug combinations, such as cladribine, idarubicin, and cytarabine (CLIA), have been shown to provide improved outcomes, especially in younger patients with AML (38).

[b]In addition to being effective in patients without mutations, venetoclax showed effectiveness in patients with *IDH1*, *IDH2*, or *FLT3* mutations.
[c]New data from the phase 3 LACEWING study support the safety and feasibility of gilteritinib plus azacitidine in patients with newly diagnosed *FLT3*-mutated AML who are ineligible for intensive induction chemotherapy, suggesting a new treatment approach for these patients (39).
[d]Based on ongoing studies in patients with newly diagnosed *IDH1*-mutated AML ineligible for intensive chemotherapy, ivosidenib is often used off-label in combination with azacitidine (40).
[e]Based on ongoing studies in patients with newly diagnosed *IDH2*-mutated AML, enasidenib is often used off-label in combination with azacitidine (41).

References

1. Röllig C, Thiede C, Gramatzki M, Aulitzky W, Bodenstein H, Bornhauser M, et al. A novel prognostic model in elderly patients with acute myeloid leukemia: results of 909 patients entered into the prospective AML96 trial. Blood (2010) 116:971-978. doi: 10.1182/blood-2010-01-267302
2. Burnett AK, Russell NH, Hills RK, Kell J, Cavenagh J, Kjeldsen L, et al. A randomized comparison of daunorubicin 90 mg/m^2 vs 60 mg/m^2 in AML induction: results from the UK NCRI AML17 trial in 1206 patients. Blood (2015) 125:3878-3885. doi: 10.1182/blood-2015-01-623447
3. Fernandez HF, Sun Z, Yao X, Litzow MR, Luger SM, Paietta EM, et al. Anthracycline dose intensification in acute myeloid leukemia. N Engl J Med (2009) 361:1249-1259. doi: 10.1056/NEJMoa0904544
4. Buckley SA, Othus M, Vainstein V, Abkowitz JL, Estey EH, Walter RB. Prediction of adverse events during intensive induction chemotherapy for acute myeloid leukemia or high-grade myelodysplastic syndromes. Am J Hematol (2014) 89:423-428. doi: 10.1002/ajh.23661
5. National Comprehensive Cancer Network. NCCN clinical practice guidelines in oncology (NCCN guidelines) - acute myeloid leukemia, version 2.2022.
6. Bishop JF, Matthews JP, Young GA, Szer J, Gillett A, Joshua D, et al. A randomized study of high-dose cytarabine in induction in acute myeloid leukemia. Blood (1996) 87:1710-1717. doi: 10.1182/blood.V87.5.1710.1710
7. Parker JE, Pagliuca A, Mijovic A, Cullis JO, Czepulkowski B, Rassam SM, et al. Fludarabine, cytarabine, G-CSF and idarubicin (FLAG-IDA) for the treatment of poor-risk myelodysplastic syndromes and acute myeloid leukaemia. Br J Haematol (1997) 99:939-944. doi: 10.1046/j.1365-2141.1997.4763281.x
8. Dinardo CD, Lachowiez CA, Takahashi K, Loghavi S, Xiao L, Kadia T, et al. Venetoclax combined with FLAG-IDA induction and consolidation in newly diagnosed and relapsed or refractory acute myeloid leukemia. J Clin Oncol (2021) 39:2768-2778. doi: 10.1200/JCO.20.03736

9. Lancet JE, Uy GL, Newell LF, Lin TL, Ritchie EK, Stuart RK, et al. CPX-351 versus 7+3 cytarabine and daunorubicin chemotherapy in older adults with newly diagnosed high-risk or secondary acute myeloid leukaemia: 5-year results of a randomised, open-label, multicentre, phase 3 trial. Lancet Haematol (2021) 8:e481-e491. doi: 10.1016/s2352-3026(21)00134-4
10. Lancet JE, Uy GL, Cortes JE, Newell LF, Lin TL, Ritchie EK, et al. CPX-351 (cytarabine and daunorubicin) liposome for injection versus conventional cytarabine plus daunorubicin in older patients with newly diagnosed secondary acute myeloid leukemia. J Clin Oncol (2018) 36:2684-2692. doi: 10.1200/JCO.2017.77.6112
11. Kubal TE, Salamanca C, Komrokji RS, Sallman DA, Sweet KL, Padron E, et al. Safety and feasibility of outpatient induction chemotherapy with CPX-351 in selected older adult patients with newly diagnosed AML. J Clin Oncol (2018) 36:e19013-e19013. doi: 10.1200/JCO.2018.36.15_suppl.e19013
12. Kolitz JE, Strickland SA, Cortes JE, Hogge D, Lancet JE, Goldberg SL, et al. Consolidation outcomes in CPX-351 versus cytarabine/daunorubicin-treated older patients with high-risk/secondary acute myeloid leukemia. Leuk Lymphoma (2020) 61:631-640. doi: 10.1080/10428194.2019.1688320
13. Talati C, Frantz D, Lubas A, Salamanca C, Tobon K, Kubal T. How I treat newly diagnosed acute myeloid leukemia in an outpatient setting: a multidisciplinary team perspective. Future Oncol (2020) 16:281-291. doi: 10.2217/fon-2019-0781
14. Deutsch YE, Presutto JT, Brahim A, Raychaudhuri J, Ruiz MA, Sandoval-Sus J, et al. Safety and feasibility of outpatient liposomal daunorubicin and cytarabine (Vyxeos) induction and management in patients with secondary AML. Blood (2018) 132:3559-3559. doi: 10.1182/blood-2018-99-115682
15. Kasner MT. Outpatient administration of liposomal daunorubicin and cytarabine (Vyxeos) in patients with secondary acute myeloid leukemia. Clin Adv Hematol Oncol (2019) 17:604-606.
16. Dombret H, Seymour JF, Butrym A, Wierzbowska A, Selleslag D, Jang JH, et al. International phase 3 study of azacitidine vs conventional care regimens in older patients with newly diagnosed AML with >30% blasts. Blood (2015) 126:291-299. doi: 10.1182/blood-2015-01-621664
17. Danilov AV, Smith H, Relias V, Miller KB. Outpatient decitabine in elderly patients with acute myeloid leukemia (AML). Blood (2009) 114:Abstract 4144. doi: 10.1182/blood.V114.22.4144.4144
18. Rybka J, Stefanko E, Bogucka-Fedorczuk A, Butrym A, Poreba R, Kuliczkowski K, et al. Azacitidine in outpatient treatment - single center experience. Contemp Oncol (Pozn) (2015) 19:467-470. doi: 10.5114/wo.2015.56653
19. Kantarjian HM, Thomas XG, Dmoszynska A, Wierzbowska A, Mazur G, Mayer J, et al. Multicenter, randomized, open-label, phase III trial of decitabine versus patient choice, with physician advice, of either supportive care or low-dose cytarabine for the treatment of older patients with newly diagnosed acute myeloid leukemia. J Clin Oncol (2012) 30:2670-2677. doi: 10.1200/JCO.2011.38.9429
20. Dinardo CD, Pratz K, Pullarkat V, Jonas BA, Arellano M, Becker PS, et al. Venetoclax combined with decitabine or azacitidine in treatment-naive, elderly patients with acute myeloid leukemia. Blood (2019) 133:7-17. doi: 10.1182/blood-2018-08-868752
21. Wei AH, Strickland SA, Jr., Hou JZ, Fiedler W, Lin TL, Walter RB, et al. Venetoclax combined with low-dose cytarabine for previously untreated patients with acute myeloid leukemia: results from a phase Ib/II study. J Clin Oncol (2019) 37:1277-1284. doi: 10.1200/JCO.18.01600

22. Dinardo CD, Jonas BA, Pullarkat V, Thirman MJ, Garcia JS, Wei AH, et al. Azacitidine and venetoclax in previously untreated acute myeloid leukemia. N Engl J Med (2020) 383:617-629. doi: 10.1056/NEJMoa2012971
23. Wei AH, Montesinos P, Ivanov V, Dinardo CD, Novak J, Laribi K, et al. Venetoclax plus LDAC for newly diagnosed AML ineligible for intensive chemotherapy: a phase 3 randomized placebo-controlled trial. Blood (2020) 135:2137-2145. doi: 10.1182/blood.2020004856
24. Pelcovits A, Moore J, Bakow B, Niroula R, Egan P, Reagan JL. Tumor lysis syndrome risk in outpatient versus inpatient administration of venetoclax and hypomethlators for acute myeloid leukemia. Support Care Cancer (2021). doi: 10.1007/s00520-021-06119-7
25. Pollyea DA, Amaya M, Strati P, Konopleva MY. Venetoclax for AML: changing the treatment paradigm. Blood Adv (2019) 3:4326-4335. doi: 10.1182/bloodadvances.2019000937
26. Jonas BA, Pollyea DA. How we use venetoclax with hypomethylating agents for the treatment of newly diagnosed patients with acute myeloid leukemia. Leukemia (2019) 33:2795-2804. doi: 10.1038/s41375-019-0612-8
27. Kadia TM, Reville PK, Wang X, Rausch CR, Borthakur G, Pemmaraju N, et al. Phase II study of venetoclax added to cladribine plus low-dose cytarabine alternating with 5-azacitidine in older patients with newly diagnosed acute myeloid leukemia. J Clin Oncol (2022):JCO2102823. doi: 10.1200/JCO.21.02823
28. Kadia TM, Cortes J, Ravandi F, Jabbour E, Konopleva M, Benton CB, et al. Cladribine and low-dose cytarabine alternating with decitabine as front-line therapy for elderly patients with acute myeloid leukaemia: a phase 2 single-arm trial. Lancet Haematol (2018) 5:e411-e421. doi: 10.1016/S2352-3026(18)30132-7
29. Stone RM, Mandrekar SJ, Sanford BL, Laumann K, Geyer S, Bloomfield CD, et al. Midostaurin plus chemotherapy for acute myeloid leukemia with a FLT3 mutation. N Engl J Med (2017) 377:454-464. doi: 10.1056/NEJMoa1614359
30. Perl AE, Martinelli G, Cortes JE, Neubauer A, Berman E, Paolini S, et al. Gilteritinib or chemotherapy for relapsed or refractory FLT3-mutated AML. N Engl J Med (2019) 381:1728-1740. doi: 10.1056/NEJMoa1902688
31. Dinardo CD, Stein EM, De Botton S, Roboz GJ, Altman JK, Mims AS, et al. Durable remissions with ivosidenib in IDH1-mutated relapsed or refractory AML. N Engl J Med (2018) 378:2386-2398. doi: 10.1056/NEJMoa1716984
32. Roboz GJ, Dinardo CD, Stein EM, De Botton S, Mims AS, Prince GT, et al. Ivosidenib induces deep durable remissions in patients with newly diagnosed IDH1-mutant acute myeloid leukemia. Blood (2020) 135:463-471. doi: 10.1182/blood.2019002140
33. Montesinos P, Recher C, Vives S, Zarzycka E, Wang J, Bertani G, et al. Ivosidenib and azacitidine in IDH1-mutated acute myeloid leukemia. N Engl J Med (2022) 386:1519-1531. doi: 10.1056/NEJMoa2117344
34. Stein EM, Dinardo CD, Pollyea DA, Fathi AT, Roboz GJ, Altman JK, et al. Enasidenib in mutant IDH2 relapsed or refractory acute myeloid leukemia. Blood (2017) 130:722-731. doi: 10.1182/blood-2017-04-779405
35. Castaigne S, Pautas C, Terre C, Raffoux E, Bordessoule D, Bastie JN, et al. Effect of gemtuzumab ozogamicin on survival of adult patients with de-novo acute myeloid leukaemia (ALFA-0701): a randomised, open-label, phase 3 study. Lancet (2012) 379:1508-1516. doi: 10.1016/S0140-6736(12)60485-1

36. Cortes JE, Heidel FH, Hellmann A, Fiedler W, Smith BD, Robak T, et al. Randomized comparison of low dose cytarabine with or without glasdegib in patients with newly diagnosed acute myeloid leukemia or high-risk myelodysplastic syndrome. Leukemia (2019) 33:379-389. doi: 10.1038/s41375-018-0312-9
37. Wei AH, Dohner H, Pocock C, Montesinos P, Afanasyev B, Dombret H, et al. Oral azacitidine maintenance therapy for acute myeloid leukemia in first remission. N Engl J Med (2020) 383:2526-2537. doi: 10.1056/NEJMoa2004444
38. Jain P, Kantarjian HM, Ravandi F, Jabbour E, Daver N, Pemmaraju N, et al. Cladribine combined with idarubicin and Ara-C (CLIA) as a frontline and salvage treatment for young patients (≤65 yrs) with acute myeloid leukemia. Blood (2016) 128:1639. doi: 10.1182/blood.V128.22.1639.1639
39. Wang ES, Montesinos P, Minden MD, Lee JH, Heuser M, Naoe T, et al. 27 Phase 3, multicenter, open-label study of gilteritinib, gilteritinib plus azacitidine, or azacitidine alone in newly diagnosed FLT3 mutated (FLT3mut+) acute myeloid leukemia (AML) patients ineligible for intensive induction chemotherapy. Presented at: 62nd ASH Annual Meeting and Exposition; December 5-8, 2020; Virtual.
40. Dinardo CD, Stein AS, Stein EM, Fathi AT, Frankfurt O, Schuh AC, et al. Mutant isocitrate dehydrogenase 1 inhibitor ivosidenib in combination with azacitidine for newly diagnosed acute myeloid leukemia. J Clin Oncol (2021) 39:57-65. doi: 10.1200/JCO.20.01632
41. Dinardo CD, Schuh AC, Stein EM, Montesinos P, Wei AH, De Botton S, et al. Enasidenib plus azacitidine versus azacitidine alone in patients with newly diagnosed, mutant-IDH2 acute myeloid leukaemia (AG221-AML-005): a single-arm, phase 1b and randomised, phase 2 trial. Lancet Oncol (2021) 22:1597-1608. doi: 10.1016/s1470-2045(21)00494-0
